# MSIF-LNP: microbial and human health association prediction based on matrix factorization noise reduction for similarity fusion and bidirectional linear neighborhood label propagation

**DOI:** 10.3389/fmicb.2023.1216811

**Published:** 2023-06-14

**Authors:** Hui Xiang, Rong Guo, Li Liu, Tengjie Guo, Quan Huang

**Affiliations:** ^1^College of Physical Education, Southwest Forestry University, Kunming, Yunnan, China; ^2^College of Physical Education, Suzhou University, Suzhou, Anhui, China; ^3^College of Physical Education, Yunnan Normal University, Kunming, Yunnan, China

**Keywords:** microbe, disease, similarity fusion, label propagation, associations prediction

## Abstract

Studies have shown that microbes are closely related to human health. Clarifying the relationship between microbes and diseases that cause health problems can provide new solutions for the treatment, diagnosis, and prevention of diseases, and provide strong protection for human health. Currently, more and more similarity fusion methods are available to predict potential microbe-disease associations. However, existing methods have noise problems in the process of similarity fusion. To address this issue, we propose a method called MSIF-LNP that can efficiently and accurately identify potential connections between microbes and diseases, and thus clarify the relationship between microbes and human health. This method is based on matrix factorization denoising similarity fusion (MSIF) and bidirectional linear neighborhood propagation (LNP) techniques. First, we use non-linear iterative fusion to obtain a similarity network for microbes and diseases by fusing the initial microbe and disease similarities, and then reduce noise by using matrix factorization. Next, we use the initial microbe-disease association pairs as label information to perform linear neighborhood label propagation on the denoised similarity network of microbes and diseases. This enables us to obtain a score matrix for predicting microbe-disease relationships. We evaluate the predictive performance of MSIF-LNP and seven other advanced methods through 10-fold cross-validation, and the experimental results show that MSIF-LNP outperformed the other seven methods in terms of AUC. In addition, the analysis of Cystic fibrosis and Obesity cases further demonstrate the predictive ability of this method in practical applications.

## 1. Introduction

With the development of biological experiment technology, more and more studies have proved that gene cells (Hu et al., 2023), drug development (Wang et al., 2023), human metabolites (Sun et al., 2022) and microbes have a certain relationship with human health. Association between small molecules, circRNA and Mirna plays a role in the treatment of human disease (Chen et al., 2020; Peng et al., 2022b; Wang C. C. et al., 2022; Wang S. H. et al., 2022). Microbes play a critical role in human health and diseases (Rastelli et al., 2018). For example, in the human gut, microbes can help synthesize various beneficial digestive enzymes (Sommer and Bäckhed, 2013), and there is a clear correlation between the occurrence and exacerbation of asthma and microbial communities (Ver Heul et al., 2019). Thus, it is important to clarify the relationship between microbes and diseases in humans. Biomedical researchers currently use traditional experimental methods to validate the potential association between microbes and diseases, but traditional experimental methods often require huge investments of time, money, and effort. Therefore, if the latest computer technologies are combined with bioinformatics methods, it is possible to efficiently obtain effective correlations between microbes and diseases. So far, to better utilize computer technologies for predicting potential microbe-disease associations, an increasing number of relevant databases have been established, including HMDAD, which records associations between diseases and microbes, and HPMCD, where users can search for microbial communities related to diseases or health, among others (Zhao et al., 2021). Association and similarity data are commonly used as inputs for prediction methods. There are two types of similarity data: those based on the original association calculation and those based on other data calculations. Using microbe and disease similarity as prior information can improve the final prediction performance of prediction methods. To date, a variety of similarity calculation methods have been proposed. Those based on the original association calculation include Gaussian, cosine, and linear neighborhood similarity, while those based on other data calculations include disease semantic similarity, symptom-based disease similarity, microbe similarity based on protein families, and functional similarity, among others (Wen et al., 2021). In recent years, with the development of computer technologies and based on existing association data and similarity calculation methods, microbe-disease prediction methods have thrived (Wang L. et al., 2022), mainly divided into three types: (1) matrix completion-based methods, (2) machine learning-based methods, and (3) network-based methods.

The first method is based on matrix completion. Matrix completion methods often use incomplete matrices to obtain a complete matrix by decomposing the known matrix and then using the decomposed matrix. Shi et al. (2018) proposed a new algorithm called BMCMDA, which established a relationship model between a parameterized matrix and a microbe-disease matrix based on known microbe-disease pairs. The algorithm inferred the likelihood of a microbe being related to a specific disease based on the recovered parameterized matrix. Wu et al. (2019) proposed an algorithm called MHMDA, which treated potential associations as unknown matrix elements and used matrix completion to predict potential microbe-disease associations. Long et al. (2021) proposed a bidirectional interaction aggregator for denoising and a learning framework that combined graph attention networks and inductive matrix completion (GATMDA). Hua et al. (2022) proposed a method, MVGCNMDA, which combines graph convolution and convolutional neural networks to compute the similarity matrix of microbe-disease associations, followed by matrix completion to predict the final results. Liu et al. (2023) constructed a heterogeneous network of microbes and diseases and used low-rank matrix factorization and nuclear norm minimization to predict the associations between microbes and diseases. However, current matrix completion-based methods are based on low-order information, often neglecting high-order information between microbes and diseases.

The second approach is based on machine learning. The rapid development of computer technology has made machine learning achieve good results in the direction of microbial disease association. Wang et al. (2017) designed a Laplacian regularized least squares classifier and developed a Laplacian regularized least semi-supervised model (LRLSHMDA) for association prediction. Peng et al. (2018) combined multiple weak classifiers into a strong classifier for prediction, proposing an adaptive boosting model (ABHMDA) for prediction. Li et al. (2020) constructed a three-layer backpropagation neural network model (BPNNHMDA) to discover potential associations. Long et al. (2021) first proposed a learning framework (GATMDA) based on graph attention network, double interaction aggregator, and inductive matrix completion. Peng et al. (2022a) integrated different similarities to construct a high-dimensional matrix, and used an autoencoder to reduce its dimensionality. A new computational method based on a deep autoencoder and an extensible tree-enhanced model (DAESTB) was proposed to predict small molecules and Potential association of miRNAs. Yu et al. (2023) using sparse relational data and finite feature data, a new graph contrast learning model based on sparse relationship enhancement and cascaded multicore fusion network (CasMF-GCL) based on machine learning is proposed. Although machine learning-based methods have performed well, the limited number of known microbe-disease association data to some extent restricts the performance of association prediction based on machine learning.

The third method is based on network approaches. Huang Y. A. et al. (2017) proposed a new computational method (NGRHMDA) which combines two single prediction models, namely the neighbor-based and graph-based prediction models, to calculate microbe-disease association prediction scores and achieve better prediction performance than single models. Huang Z. A. et al. (2017) proposed a method based on known similarities, using a deep traversal method to explore the potential path between microbes and diseases, so as to obtain the potential associations between microbes and diseases. Wang et al. (2021) proposed a new computational model, named MSLINE, to infer potential microbe-disease associations by combining multiple similarity and large-scale information network embedding (LINE) based on known associations. Chen et al. (2021) construct a Heterogeneous Network for Small Molecule-miRNA Using Bounded Kernel Canonical Regularization to Predict (SM-miRNA) Association Prediction (BNNRSMMA). Yin et al. (2023) proposed a method based on two-layer double random walks to combine different microbial and disease similarity networks (NTBiRW), and finally calculated the final prediction score based on K-nearest neighbors. Jiang et al. (2018) proposed a new multi-similarity kernel fusion method (SKF) in MDA-SKF to study the correlation between LncRNA and disease, and used a weighted matrix to denoise the fused matrix. Although this method uses a weighted method for denoising, the information of the similar network itself is still lost in the fusion process, resulting in a decrease in prediction accuracy due to the lack of original node similarity information. In order to solve the problem of information loss during the fusion process of the self-network nodes, Xie G. B. et al. (2023) proposed a method of adding a unit matrix in the process of similarity matrix fusion, which keeps the original similarity while cutting down on noise throughout the fusion process, but does not fundamentally solve the noise problem. Therefore, we propose a matrix decomposition (SVD)-based method to extract key information after fusion matrix, further improving the denoising effect.

In order to overcome the problem of noise in the fusion of similar networks in microbe-disease association prediction, we developed a new method called MSIF-LNP, which combines MSIF and linear neighborhood label propagation (LNP) to predict microbe-disease associations. MSIF-LNP can predict association scores between microbes and diseases from three directions: Gaussian similarity, cosine similarity, and linear neighborhood similarity. We constructed two similar networks of microbes and diseases in MSIF through nonlinear cross-iteration, using the method of neighbor matrix-weighted constraint kernel, and denoised the fusion matrix using matrix factorization (SVD). To obtain the final prediction results of microbe-disease association, we used LNP to propagate the initial microbe-disease association information as labels on the two constructed microbes and disease networks. The MSIF-LNP model was validated using 10-fold cross-validation (10-fold-CV), and the validation results showed that the performance of MSIF-LNP was superior to the other seven microbe-disease prediction algorithms. In addition, among the top 10 expected microbes for the respective diseases (Cystic fibrosis and Obesity), nine were confirmed in case studies.

## 2. Materials and methods

### 2.1. Datasets

The selection of a well-known and reliable microbe-disease association dataset is a crucial step to make the accuracy of the established prediction model as accurate as possible. We selected HMDAD,^1^ a microbes disease dataset, and merged the microbe-disease associations collected in it. Finally, we obtained 450 experimentally validated microbe-disease associations with 292 microbes and 39 diseases, respectively.

### 2.2. Cosine similarity

Cosine similarity is a commonly used similarity measure (Xia et al., 2015), which measures the similarity between two vectors in a vector space based on their cosine angle. In microbes space, we calculate the cosine similarity between microbes vectors using a known microbe-disease association matrix, which is divided into two main steps. First, we use $P\left(m_{i}\right)$ to denote the microbes $m_{i}$ relationship vector with each disease, where $m_{i}$ refers to the i-th row of the microbe-disease association matrix. In the second step, we calculate the cosine similarity of each microbe pair is calculated using P(microbes) to the microbe $m_{i}$ with microbe $m_{j}$ as an example, the cosine similarity formula can be expressed as:


(1) $$COS\_M\left(m\_i,m\_j\right)=\frac{P\left(m\_i\right)\cdot P\left(m\_j\right)}{\left(∥P\left(m\_i\right)∥*∥P\left(m\_j\right)∥\right)}$$

$COS_{M}\left(m_{i},m_{j}\right)$ indicates microbe $m_{i}$ and microbe $m_{j}$ of the cosine similarity value, the symbol·indicates the vector dot product operation. After calculation, the cosine similarity of all microbes pairs forms the microbes similarity matrix $COS_{M}$. By the same token, we can calculate the cosine similarity matrix $COS_{D}$ of the diseases.

### 2.3. Gaussian similarity

To diversify the similarity information between microbes data, we introduced Gaussian interaction similarity to calculate the degree of similarity between microbes, and constructed a kernel similarity matrix of microbes Gaussian interaction properties using a known microbe-disease association relationship matrix. For microbes data, microbe $m_{i}$ and microbe $m_{j}$ the Gaussian interaction property kernel similarity formula is as follows.


(2) $$GM\left(m_{i},m_{j}\right)=\exp\left(-\gamma_{m}||A\left(m_{i},:\right)-A\left(m_{j},:\right)|{|}^{2}\right)$$

(3) $$\gamma_{m}=\gamma /\left(\frac{1}{nm}\sum_{i=1}^{nm}||A\left(m_{i},:\right)|{|}^{2}\right)$$

where A denotes a known microbe-disease association, $\gamma_{m}$ is the bandwidth used to control the kernel similarity of Gaussian interaction properties and the value of γ is generally 1, nm is the number of microbes. By the same token, we can derive the disease Gaussian interaction property kernel similarity matrix GD.

### 2.4. Linear neighborhood similarity

We use linear domain similarity based on linear domain data to calculate this prediction model (Zhang et al., 2018). For the microbes data, it is assumed that $t_{i}$ represents the feature vector of the i-th microbe, and our objective is to minimize.


$$\delta =t_{i}-{\left\|\sum_{i_{j}:t_{i_{j}}\in N\left(t_{i}\right)}w_{ii_{j}}t_{i_{j}}\right\|}^{2}$$

(4) $$s.t.\;\;\sum_{i_{j}:t_{i_{j}}\in N\left(t_{i}\right)}w_{ii_{j}}=1,\;\;w_{ii_{j}}≧0$$

where $N\left(t_{i}\right)$ denotes $t_{i}$ the N (free parameter) nearest neighbor set (*via* Euclidean distance), the $t_{i_{j}}$ is the $t_{i}$ the jth neighbor, and the $w_{ii_{j}}$ is the measure of $t_{i_{j}}$ the contribution to the $t_{i}$ the reconstruction contribution, which can be used as a similarity metric. Using quadratic programming to solve the equation, we get $t_{i}$ the linear neighborhood reconstruction weights of the domain, for any $t_{j}\notin N\left(t_{i}\right)$ that $w_{ii_{j}}=0$. One way to assess similarity is to look at a data point’s neighbors’ weights. Thus, we are able to get the linear neighborhood similarity matrix LM between microbes. In a similar vein, we can also get the linear neighborhood similarity matrix LD between diseases.

### 2.5. MSIF-LNP method

#### 2.5.1. Overview

**Figure. 1:**
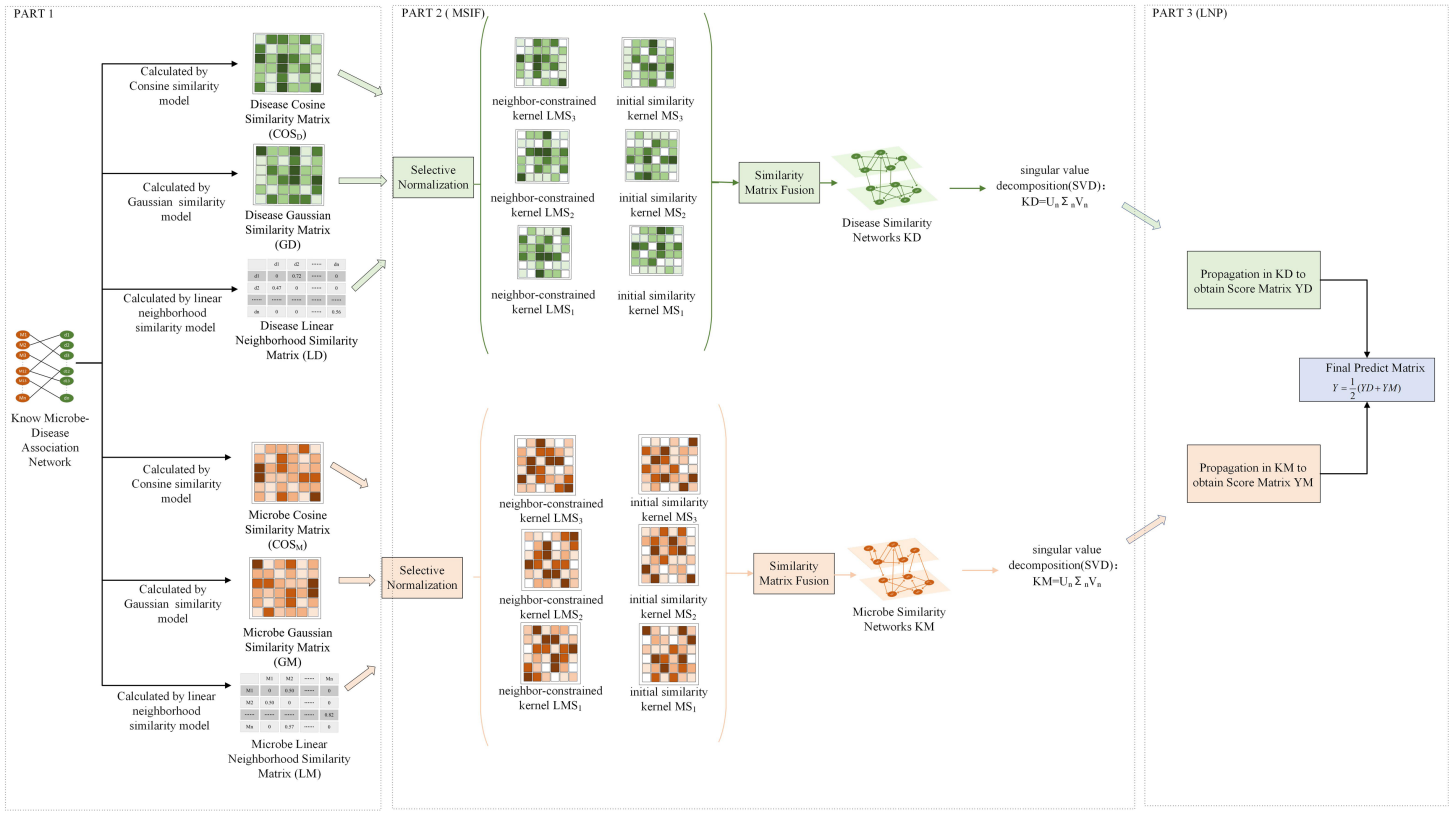
Flow chart of MSIF-LNP.

In this study, MSIF-LNP was utilized to predict the possible relationships between microbes and diseases. In MSIF, multiple initial similarity matrices were fused into a network, and then the resulting network was subjected to SVD noise reduction and the initial microbe-disease associations were used as markers for bidirectional linear domain label propagation on the network constructed in LNP. The MSIF-LNP model’s diagram, which is shown in Figure 1, has three parts: data processing, MSIF, and LNP.

#### 2.5.2. Similarity matrix noise reduction fusion (MSIF)

To be able to more accurately combine the numerous parallels between the microbes and diseases above and to reduce the noise generated by fusing similarity matrices, we have adopted a similarity matrix noise reduction fusion method.

In order to better similarity fusion, we must preprocess the three initial similarity matrices by bi-directional selective normalization of the matrices (Xie G. B. et al., 2023), which allows us to exclude the effect of all-zero rows and columns on the model and enhance robustness. Additionally, three neighborhood constraint kernels must be created for the three disease/microbe similarity matrices and selectively normalize the three kernels. The selective normalization method for creating the initial similarity kernels is the following:


$$MS_{1}=\frac{COS_{M}\left(m_{i},m_{j}\right)}{\sum_{m_{i}\in M}COS_{M}\left(m_{i},m_{j}\right)}$$

(5) $$s.t.\underset{m_{i}\in M}{\sum }COS_{M}\left(m_{i},m_{j}\right)\neq 0$$

where $COS_{M}$ denotes the microbes cosine similarity matrix, M denotes 292 microbes, and $MS_{1}$ denotes the initial kernel of microbes expression similarity after column normalization, satisfying $\sum_{m_{i}\in M}MS_{1}\left(m_{i},m_{j}\right)\neq 0,$1. Where $\sum_{m_{i}\in M}COS_{M}\left(m_{i},m_{j}\right)\neq 0,$ indicates that after the column normalization is complete, the column in the expression similarity matrix with all zeros is not chosen. In the same principle, we can also obtain the corresponding initial similarity kernels by column normalizing the microbes Gaussian similarity matrix GM and the microbes linear domain similarity matrix LM, respectively $MS_{2}$ and $MS_{3}$.

The formula for constructing the selective row normalization of the neighborhood constraint kernel is as follows.


$$LMS_{1}=\left\{\begin{array}{l} \frac{COS_{M}\left(m_{i},m_{j}\right)}{\sum_{m_{j}\in M}COS_{M}\left(m_{i},m_{j}\right)},\;if\;m_{j}\in N_{m}\\ 0\;,\;if\;m_{j}\notin N_{m} \end{array}\right.$$

(6) $$s.t.\;\;\sum_{m_{j}\in M}COS_{M}\left(m_{i},m_{j}\right)\neq 0$$

where $LMS_{1}$ is the domain constraint kernel with row normalization of the microbes cosine similarity matrix and satisfies $\sum_{m_{j}\in M}LMS_{1}\left(m_{i},m_{j}\right)=1$. $N_{i}$ is the set of neighbors corresponding to microbe i (including itself), while the number of neighbors of microbe i is $N_{m}$. In a similar vein, we may determine the neighbor constraint kernels for the microbe Gaussian similarity matrix GM and the microbe linear domain similarity matrix LM, respectively $LMS_{2}$ and the $LMS_{3}$.

After the above series of treatment, three initial similarity kernels are obtained $MS_{l},l=1,2,3\;$and the neighbor constraint kernel $LMS_{l},l=1,2,3$. We propose a similar kernel fusion method to fuse the initial similar kernel and the adjacent constraint kernel, the relevant formula is as follows.


(7) $$LMS_{l}^{x+1}=MS_{l}\times \frac{\sum_{r\neq l}LMS_{r}^{x}}{2}\times MS_{l}^{T}$$

Which $LMS_{l}^{x+1}$ is the number of iterations after the x+1 after the first iteration of the l state of the first kernel, and $MS_{l}^{T}$ represents the $MS_{l}$ is the transpose matrix of After x+1 iterations, the similarity network of microbe KM can be expressed as


(8) $$KM=\frac{1}{3}\overset{3}{\underset{l=1}{\sum }}LMS_{l}^{x+1}$$

Finally, due to the matrix KM of high dimensionality, there may be noise in the matrix, therefore, we used the SVD algorithm to noise reduce the matrix and improve the quality of the data. By keeping the first n maximum singular values to reconstruct KM, the final noise-reduced microbe similarity network is obtained KM.


(9) $$KM=U_{n}\Sigma_{n}V_{n}$$

where U denotes the left singular vector matrix, Σ denotes the diagonal matrix of singular values, V denotes the right singular vector matrix, and n denotes the maximum singular value. And, we adjusted to half of the number of singular values proposed by Franceschini et al. (2016), this is because if the obtained ranking is set too low, the key data may be removed.

In the same way, according to the above steps, the disease cosine similarity KD after noise reduction can be obtained by calculated by $COS_{D}$, GD and LD.

#### 2.5.3. Bidirectional linear neighborhood label propagation (LNP)

We carry out linear domain label propagation on the created microbe similar network KM and disease similar network KDrespectively. The final forecast outcome is the average value of the prediction score obtained from the propagation in the microbes and diseases network. Since the label propagation is not purely carried out on a network, the prediction value thus obtained will be more accurate.

In LNP, we used the known microbe-disease association matrix as a marker $H^{0}$ that is propagated in the microbes/diseases similarity network (Xie G. B. et al., 2023). The label of each node at each step is obtained from the probability θ of the directed graph KM /KD of its neighbors and retains its initial labeling information at 1−θ rate of the update until the convergence propagation process can be expressed as.


(10) $$H^{m+1}=\theta *K*H^{m}+\left(1-\theta \right)H^{0}$$

The following conclusions can be drawn.


$$\underset{m\to \infty }{\lim}H^{m}=\underset{m\to \infty }{\lim}{\left(\theta *K\right)}^{m}H^{0}+\left(1-\theta \right)\overset{m-1}{\underset{i=0}{\sum }}{\left(\theta *K\right)}^{i}H^{0}$$

(11) $$=\left(1-\theta \right){\left(I-\theta *K\right)}^{-1}H^{0}$$

when linear domain labeling is performed on a microbe similarity network, the K is equal to KM, the final prediction score matrix can be derived YM:


(12) $$YM=\left(1-\theta \right){\left(I-\theta *KM\right)}^{-1}H^{0}$$

when linear domain labeling is performed on the disease similarity network, the K is equal to KD, the final prediction score matrix can be derived YD:


(13) $$YD=\left(1-\theta \right){\left(I-\theta *KD\right)}^{-1}H^{0}$$

The scoring matrix is then most normalized so that microbe-disease pairs that are associated have higher scores and microbe-disease pairs that are not associated have lower scores, and the final prediction accuracy of the model can be improved by this step, and the scoring matrix is YM The process of performing the most-valued normalization is shown below.


(14) $$YM\left(m_{i},m_{j}\right)=\frac{YM\left(m_{i},m_{j}\right)-YM_{\min}\left(m_{i},:\right)}{YM_{\max}\left(m_{i},:\right)-YM_{\min}\left(m_{i},:\right)}$$

where $YM_{\max}$ represents the maximum value of $YM\left(m_{i},:\right)$,and $YM_{\min}$ is the minimum value of $YM\left(m_{i},:\right)$.Similarly, we use the normalization to the scoring matrix YD to obtain the final scoring matrix YD. Finally, we average the two prediction score matrices and take the result as the final prediction score Y.


(15) $$Y=\frac{1}{2}\left(YM+YD\right)$$

### 2.6. Assessment indicators

In the validation process of this paper, we used the 10-flod-CV validation method to test the performance of the model. In 10-flod-CV, each known microbe-disease associations will be randomly divided into 10 groups, each group will become a test group, and the other groups will be training groups. We used the area under the curve (AUC) evaluation criterion as a performance judgment criterion for evaluating the model, and AUC is the area under the ROC curve enclosing the horizontal axis (Wang W. et al., 2022).

### 2.7. Optimal parameter selection

**Figure. 2:**
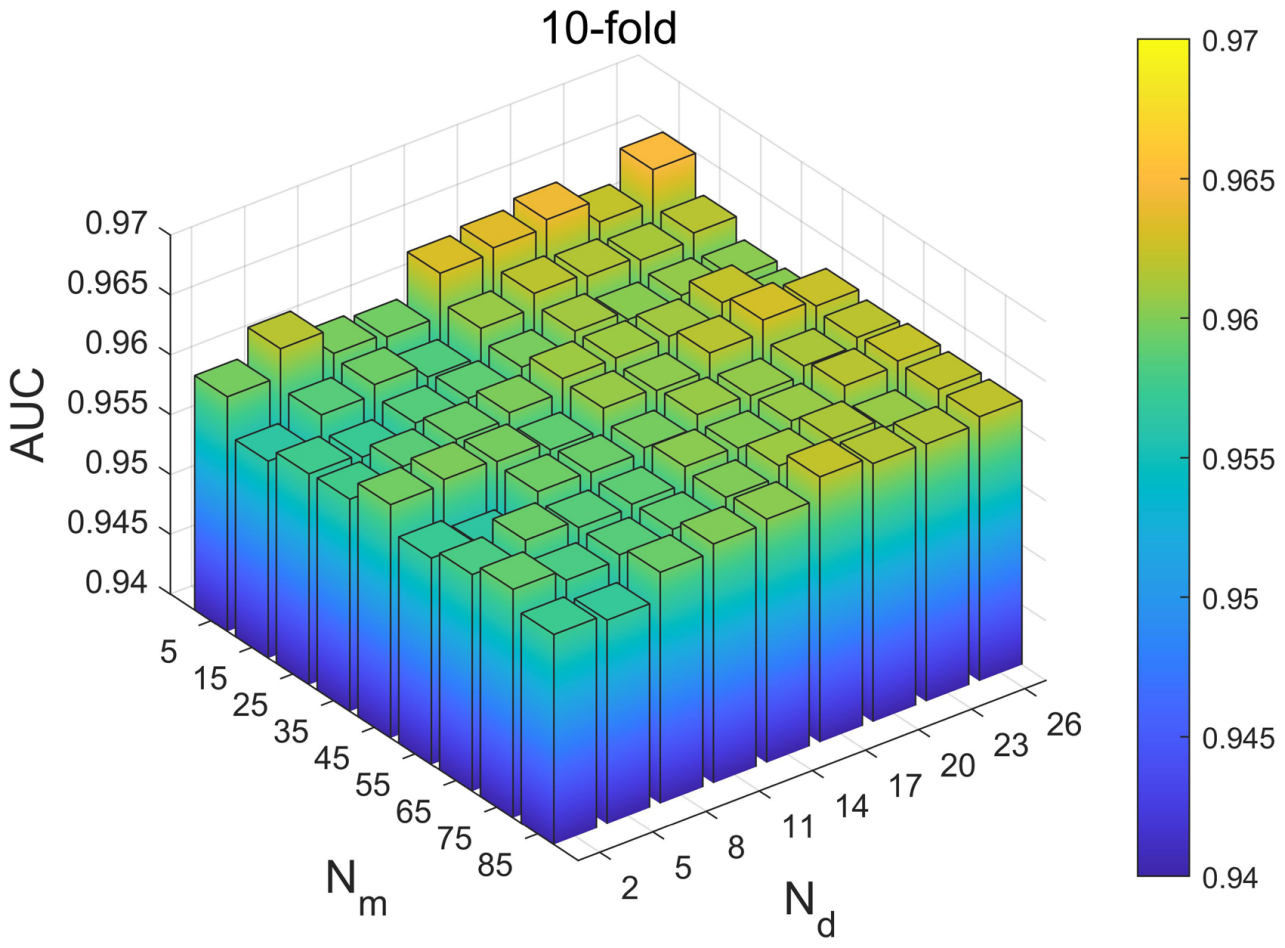
10-flod-CV under different $N_{m}$ and $N_{d}$ effects on AUC values.

In our model, it is necessary to take into account the choice of four parameters, which are $N_{m}$,$N_{d}$,x and θ.$N_{m}$ and $N_{d}$ are the number of neighbors of microbes and diseases, and through several parameter debugging, we set $N_{m}$ the selection range of $N_{m}$ =5–85, and set the $N_{d}$ the selection range of $N_{d}$ = 2–26. From Figure 2, it can be seen that the range when $N_{m}$ and $N_{d}$ are 5 and 26, respectively, the value of 10-fold-CV is the largest, and the final determination of $N_{m}$ and $N_{d}$ of the optimal parameters are 5 and 2.

**Figure. 3:**
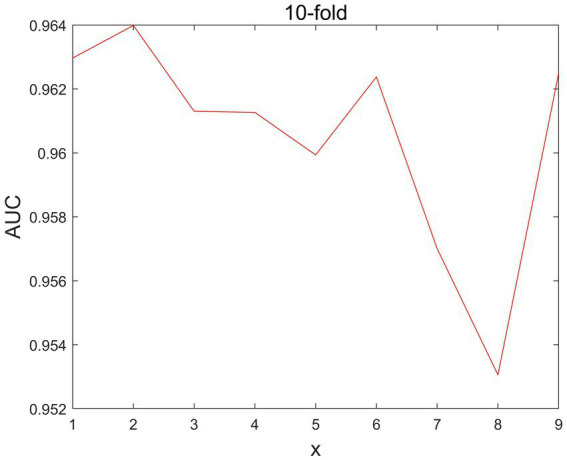
The different 10-flod-CV *x* effect on AUC values.

In MSIF, there is a parameter x is the number of iterations, and we set the number of iterations x in the range of [1,2,3,4,5,6,7,8,9]. As shown in Figure 3, the parameter debugging can be obtained when x =2, the value of AUC is the largest, so x the best parameter choice is 2.

**Figure. 4:**
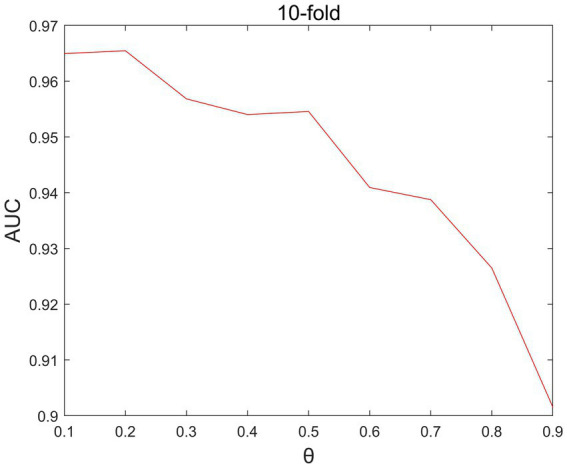
10-flod-CV under different *θ* effect on AUC values.

In the LNP, there is a parameter θ that θ is the propagation probability parameter. We set the θ the range of values of is set as [0.1,0.2,0.3,0.4,0.5,0.6,0.7,0.8,0.9]. Through parameter debugging, as shown in Figure 4, it can be seen that when *θ* =0.2, the performance of the model is best.

Based on the aforementioned tests, we ultimately identified these four parameters’ ideal values as $N_{m}$ =5, the $N_{d}$ =26, x = 2, and *θ* =0.2.

### 2.8. Algorithm comparison

**Figure. 5:**
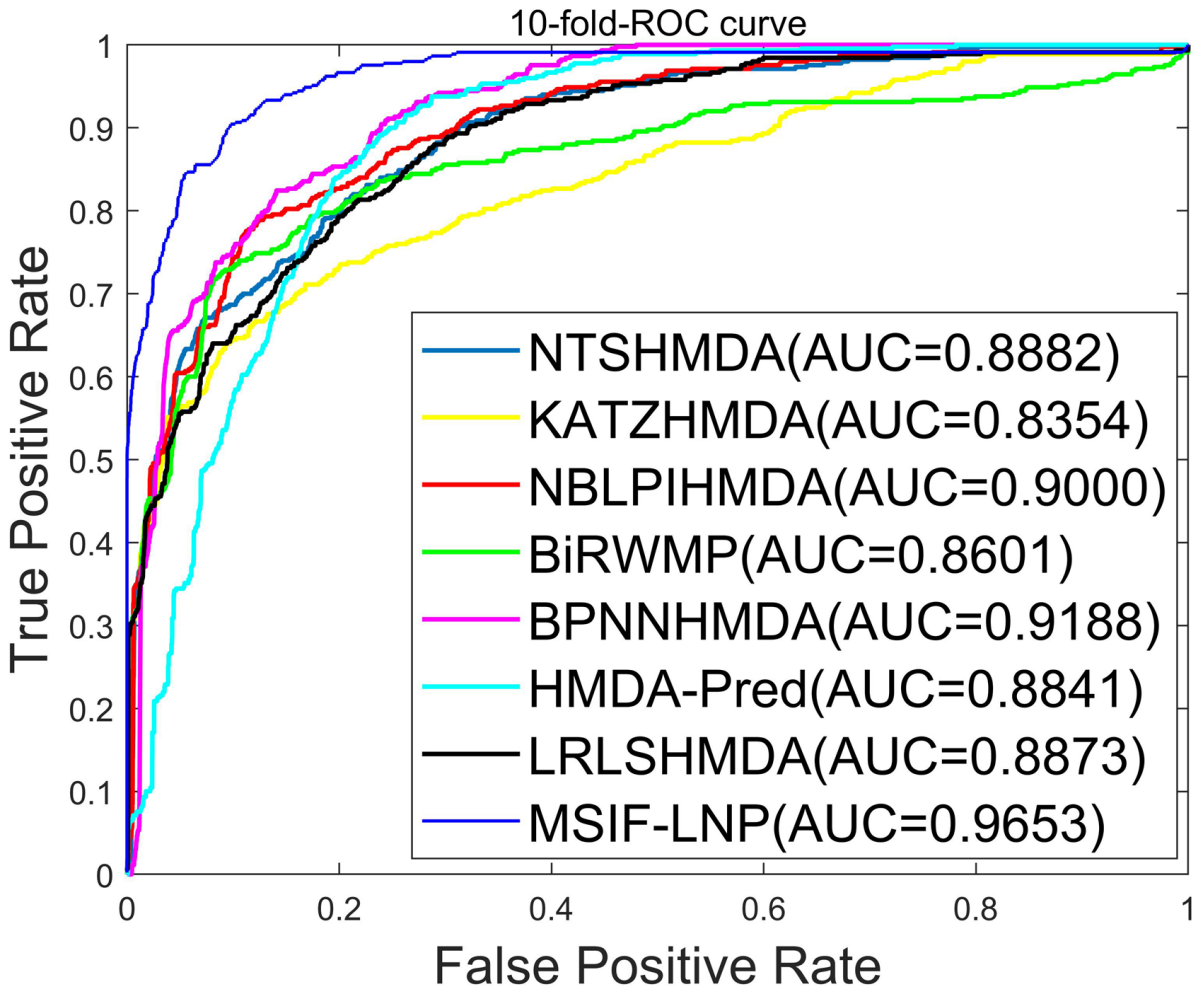
AUC of MSIF-LNP with seven other models of 10-fold-CV.

To validate the reliability of the MSIF-LNP model, we compare MSIF-LNP with NTSHMDA (Luo and Long, 2018), KATZHMDA (Chen et al., 2017), NBLPIHMDA (Wang et al., 2019), BiRWMP (Shen et al., 2018) BPNNHMDA (Li et al., 2020), HMDA-Pred (Fan et al., 2020) and LRLSHMDA (Wang et al., 2017) were compared with seven prediction methods. The comparison results under 10-flod-CV are shown in Figure 5. The maximum AUC of MSIF-LNP is 0.9653 ± 0.0002, and the values of NTSHMDA, KATZHMDA, NBLPIHMDA, BiRWMP, BPNNHMDA, HMDA-Pred and LRLSHMDA are 0.8882 ± 0.0009, 0.8354 ± 0.0033, 0.9000 ± 0.0027, 0.8601 ± 0.0089, 0.9188 ± 0.0009, 0.8841 ± 0.0037 and 0.8873 ± 0.0029, respectively. The results indicate that our method achieves better prediction than other of these methods achieved better prediction.

We performed a statistical test at the significance level $p^{\prime }=0.05$. If $p^{\prime }>p$, it means that the original hypothesis was not rejected, and there is no difference in prediction performance. If $p^{\prime }<p$, it means that the original hypothesis is rejected and there is a significant difference in the prediction algorithm (Xie G. et al., 2023). According to Table 1, we can reject the original hypothesis that the other seven models performed on the same data set have the same effect as MSIF-LNP because the p-values are all smaller than $p^{\prime }$.

**Table 1:** The statistical test between MSIF-LNP and the other seven models.

Model	NTS-HMDA	KATZH-MDA	NBLPIH-MDA	BiRW-MP	BPNNH-MDA	HMDA-Pred	LRLSH-MDA
p-value	$2.94\times 10^{-5}$	$2.56\times 10^{-14}$	$3.27\times 10^{-5}$	$1.07\times 10^{-41}$	$1.67\times 10^{-41}$	$4.38\times 10^{-11}$	$1.43\times 10^{-42}$

### 2.9. Case study

We use MSIF-LNP to predict each unrecognized microbe-disease pair and rank the resulting association prediction scores in descending order. We have selected cystic fibrosis and obesity as our case study, and we have validated the top 10 microbes in the PubMed database that rank in the prediction of these two diseases, both of which have an accuracy of 90%, and Table 2 shows the prediction results for cystic fibrosis (*CF*). *CF* is an inherited exocrine gland disease that primarily affects the gastrointestinal and respiratory systems and can lead to bronchitis, malnutrition, and other symptoms, and it is estimated that there are about 70,000 cases worldwide, with approximately 1,000 new cases added each year (Jean-Pierre et al., 2023). According to previous studies, a relationship exists between *CF* and *S. aureus*, and Jean-Pierre et al. (2023) concluded that *S. aureus* is the main opportunistic pathogen in *CF* patients and that biofilm production is a determining factor for *CF* patients to have persistent episodes of *S. aureus* respiratory infections. According to Menetrey et al. (2021), it was shown that *Stenotrophomonas maltophilia* (SM) is an emerging pathogen that shares important pathophysiological features with *CF* pathogens and that SM possesses a large number of adaptive strategies to persist in *CF* patients. In terms of exercise and health, Williams (2016) found that cystic fibrosis (*CF*) patients who exercised for more than 30 min per day had fewer hospitalizations and improved lung function after 12 months compared to *CF* patients who exercised for less than 30 min per day. Kalamara et al. (2021) also suggested that daily exercise can improve aerobic capacity and slow down the decline of lung function in *CF* patients, and a combination of aerobic and anaerobic training may be the best training approach for *CF* patients.

**Table 2:** The predicted top10 potential microbes for cystic fibrosis by MSIF-LNP.

Rank	Microbes	Evidence (PubMed ID)
1	*Staphylococcus aureus*	36614040
2	*Stenotrophomonas maltophilia*	33919046
3	Burkholderia	32239690
4	Lysobacter	18077362
5	Rickettsiales	18077362
6	*Streptococcus mitis*	31759908
7	Xanthomonas	9616541
8	Lactobacillus	30741841, 33058577
9	Coxiellaceae	unknown
10	Pseudomonas	30500353

Table 3 shows the association results of obesity-related microbes. Gut microbiota is considered an important factor in the development of metabolic diseases such as obesity, as well as an endocrine organ that maintains energy homeostasis and human immunity. Gomes et al. (2018) found that dysbiosis could alter the function of the gut barrier and gut-associated lymphoid tissue (GALT) by allowing bacterial structural components such as lipopolysaccharides (LPS) to activate inflammation in the human body, leading to increased insulin resistance. Xie et al. (2022) demonstrated through experiments that excessive neutral lipids were stored in greatly expanded lipid droplets (LDs) due to enhanced endoplasmic reticulum (ER)-LD interaction. Campbell and Wisniewski (2017) showed in their research that exercise could enhance microbial diversity, prevent weight gain, and improve body composition, such as reducing fat mass, in the context of a high-fat diet (HFD). However, the effects of exercise are not limited to increasing diversity. Exercise can also reduce inflammatory mediators, increase antioxidant enzymes, and reduce the expression of tumor necrosis factor (TNF)-α in gut lymphocytes. Exercise can promote gut health and microbial diversity, thereby reducing the risk of chronic diseases.

**Table 3:** The predicted top10 potential microbes for obesity by MSIF-LNP.

Rank	Microbes	Evidence (PubMed ID)
1	*Staphylococcus aureus*	29667480
2	*Alcaligenaceae*	unknown
3	*Coriobacteriaceae*	29152632
4	*Erysipelotrichaceae*	34053553
5	*Methanobrevibacter smithii*	32231226
6	*Prevotellaceae*	29434314
7	*Firmicutes*	29667480
8	*Bacteroidetes*	32438689
9	*Proteobacteria*	26102296
10	*Stenotrophomonas maltophilia*	35112996

## 3. Conclusion

Microbes are closely related to human health and have played important roles in drug development, medical beauty, disease diagnosis and treatment, and other fields. In order to understand the relationship between microbes and human health, it is necessary to clarify the potential relationship between microbes and diseases. To this end, we propose a method called MSIF-LNP to predict the potential association between microbes and diseases. After combining multiple similarity matrices and performing matrix decomposition, the noise impact of matrix fusion is reduced. Then, linear neighborhood label propagation is performed under the fused microbe/disease similarity network to obtain the final microbe-disease association score matrix. In experiments, the AUC value of MSIF-LNP in 10-fold CV was 0.9653, which was significantly better than the seven existing methods for predicting microbe-disease relationships. Meanwhile, it has been demonstrated through McNemar’s test that there are differences between MSIF-LNP and other comparative algorithms. In addition, the MSIF-LNP method was applied to case studies of cystic fibrosis and obesity, and the top 10 microbes obtained from our method were compared with clinical results. The results showed that the identification accuracy of both diseases was 90%. Therefore, MSIF-LNP has performed exceptionally well in predicting the correlation between microbes and diseases, and subsequently predicting the correlation between microbes and health. In future work, we can use other relevant information, such as genetic information between microbes, to improve the problem of matrix sparsity caused by incomplete datasets. We believe that by using the biological characteristics of microbes and human-made predictions, we can promote the development of microbes and human health for the benefit of human health and human life.

## Data availability statement

The original contributions presented in the study are included in the article/supplementary material, further inquiries can be directed to the corresponding author.

## Author contributions

HX, RG, LL, TG, and QH contributed to the conception and design of the study. HX writes the model code. RG was analyzed experimentally. LL wrote the first draft of the manuscript. XH, RG, TG, and QH wrote parts of the manuscript. All authors contributed to manuscript revisions, read and approved the submitted version.

## Conflict of interest

The authors declare that the research was conducted in the absence of any commercial or financial relationships that could be construed as a potential conflict of interest.

## Publisher’s note

All claims expressed in this article are solely those of the authors and do not necessarily represent those of their affiliated organizations, or those of the publisher, the editors and the reviewers. Any product that may be evaluated in this article, or claim that may be made by its manufacturer, is not guaranteed or endorsed by the publisher.

## Footnotes

^1^ http://www.cuilab.cn/hmdad
